# Differential expression of mitomiRs in pancreatic islet cells associated with maternal protein restriction

**DOI:** 10.1080/19382014.2025.2610590

**Published:** 2026-01-01

**Authors:** Cecile Jacovetti, Romano Regazzi

**Affiliations:** a Department of Fundamental Neurosciences, University of Lausanne, Lausanne, Switzerland; b Department of Biomedical Sciences, University of Lausanne, Lausanne, Switzerland

**Keywords:** Small non-coding RNAs, Mitochondrial non-coding transcriptome, mitomiRs, Diabetes susceptibility

## Abstract

**Objective:**

Mitochondria are central to energy production and cellular homeostasis. Beyond importing diverse RNAs, they also encode hundreds of their own non-coding RNAs, contributing to a complex and dynamic RNA landscape. Early-life nutritional insults, such as fetal and postnatal protein deficiency, can impair mitochondrial function and increase the long-term diabetes risk. However, the mitochondrial non-coding transcriptome of pancreatic islets, particularly its responsiveness to nutritional cues, remains largely unexplored.

**Methods:**

We performed RNA sequencing to profile small non-coding RNAs in mitochondrial fractions of islet cells from offspring of rats exposed to low-protein (LP) or control diets during gestation and lactation and employed mRNA-miRNA network analysis to explore the potential regulatory roles of differentially expressed mitomiRs in LP-exposed pups.

**Results:**

Protein deficiency during gestation and lactation led to a profound remodeling of the small non-coding RNA landscape in whole islets, with microRNAs and piRNAs showing the most pronounced changes. In mitochondrial fractions, LP exposure resulted in a striking shift in microRNA composition, with 33 mitomiRs detected in control islets versus 23 in LP-exposed rats, and only 5 shared between groups. Notably, ten mitomiRs were selectively depleted from the cytosol and enriched in mitochondria of LP-exposed islets. Amongst these, miR-10a-5p and miR-126a-5p, are predicted to target genes involved in mitochondrial metabolism and structural organization.

**Conclusion:**

Early-life protein restriction triggers a highly selective reorganization of the mitomiR landscape in pancreatic islets. The identified mitomiRs may serve as regulators of mitochondrial function and intracellular signaling, potentially influencing *β*-cell metabolic coupling and contributing to diabetes susceptibility.

## Graphical abstract

**Figure:**
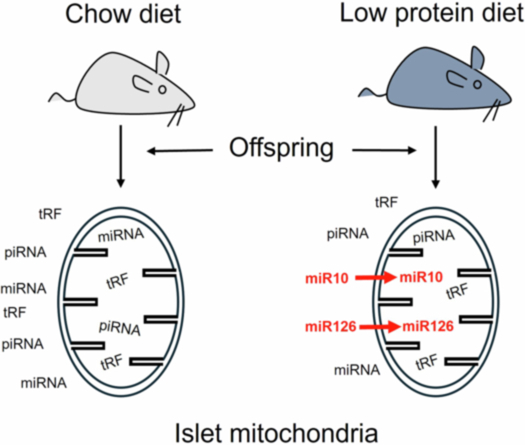

## Introduction

Pancreatic *β*-cells serve as glucose sensors, releasing insulin in tightly regulated amounts in response to rising blood glucose levels. Glucose–stimulated insulin secretion (GSIS) depends primarily on mitochondrial oxidative phosphorylation for ATP production.^1^ Mitochondrial function in *β*-cells is finely tuned by an intricate interplay of metabolic cues, enzymatic pathways, and transcriptional regulators to meet the energy demands of insulin secretion.^2^ Disruption of this energy–dependent process is a central contributor to *β*-cell failure and the onset of Type 2 Diabetes (T2D).

Although mitochondria possess their own genome, they rely heavily on continuous anterograde communication with the nucleus for import of nuclear–encoded proteins and non–coding RNAs (ncRNAs), which are essential for the function and homeostasis of these organelles. MicroRNAs are small non–coding RNAs that act as key post–transcriptional regulators, primarily by inhibiting translation or promoting degradation of target mRNAs.^3^ Their regulatory role has been extensively documented in both physiological and pathological contexts.^4^

In recent years, a distinct subset of microRNAs associated with mitochondria, termed mitomiRs, has emerged as critical for maintaining mitochondrial function and homeostasis.^5^^,^^6^ Encoded by the nuclear genome, mitomiRs can regulate mitochondrial activity either indirectly by targeting cytoplasmic mRNAs involved in mitochondrial processes or directly by localizing to the organelle. While the existence of mitochondrially–encoded microRNAs was long debated, recent advances in next–generation sequencing have revealed a diverse mitochondrial ncRNA repertoire, including long ncRNAs, circular RNAs, and microRNAs.^7-10^ The identification of Argonaute 2, a central component of the RNA–induced silencing complex, within mitochondria further supports the existence of functional post–transcriptional regulation within the organelle.^5^^,^^11^ MitomiRs are likely to play a role in sensing and responding to changes in mitochondrial microenvironment to fine–tune organelle activity.

Maternal malnutrition, particularly protein restriction during gestation, has been strongly linked to long–term metabolic dysfunction in offspring. Low–protein (LP) diets during pregnancy have been shown to impair mitochondrial function in pancreatic islets, contributing to diabetes susceptibility.^12^ Offspring of LP–fed mother exhibit altered pancreatic development, characterized by reduced *β*-cell mass and impaired GSIS. Although they initially display enhanced insulin sensitivity, this state transitions to insulin resistance and glucose intolerance in adulthood.^13^^,^^14^ Mitochondrial dysfunction in this context has been attributed in part to dysregulated expression of ncRNAs, including microRNAs and long non–coding RNAs, which affect *β*-cell proliferation and insulin secretion.^15^^,^^16^ Additionally, LP exposure alters the mitochondrial import of nuclear–encoded tRNA–derived fragments (tRFs), which are essential for mitochondrial respiration and *β*-cell function.^17^ We also previously identified a mitochondrially–encoded tRF, mt–tRF–Leu^TAA^, as a key regulator of mitochondrial metabolism and insulin secretion. This tRF is downregulated in both pancreatic islets and skeletal muscle of LP–exposed, diabetes–prone rats.^18^^,^^19^ Despite these findings, the broader dynamics and functions of mitochondrial ncRNAs in response to nutritional challenges remain poorly understood.

In this study, we aimed to characterize the small ncRNA landscape within mitochondrial fractions (MF) of pancreatic islet cells from offspring of protein–restricted versus controls dams. We further explored the biological relevance of differentially expressed mitomiRs using targetome analysis. Our findings reveal that gestational and postnatal protein deficiency leads to a striking reorganization of the mitochondrial microRNA pool. We identified a subset of ten mitomiRs that were concurrently depleted from the cytoplasm and enriched within mitochondria of LP–exposed islets. Several of these mitomiRs have known roles in *β*-cell function, suggesting that their selective mitochondrial localization may represent an adaptive or maladaptive regulatory mechanism in response to early–life nutritional stress.

Altogether, these data suggest that mitomiRs constitute an underappreciated regulatory layer of *β*-cell mitochondrial activity. By modulating mitochondrial metabolism and gene expression in response to nutritional cues, mitomiRs may influence long–term susceptibility to T2D. Further elucidation of the mitochondrial non–coding transcriptome may uncover novel therapeutic targets for metabolic diseases.

## Materials and methods

### Experimental models

Sprague Dawley rats were housed in groups of four per cage, with the exception of pregnant females, which were individually housed. They were maintained on a 12-hour light/dark cycle under controlled environmental conditions, with temperatures kept between 20-24 °C and humidity levels ranging from 40-70%. Unless they receive specific diets, the animals had free access to water and standard laboratory chow. All experimental procedures adhered to the guidelines established by the National Institutes of Health and received approval from both the National Health and Medical Research Council of Australia and the Swiss research council and veterinary authorities (permits VD2495 and VD2824).^19^ Animals were sourced from Janvier Laboratories (Le Genest–Saint–Isle, France).

Pregnant Sprague Dawley rats were provided with either a low–protein diet (5.3% protein, 17% fat, 77.7% carbohydrate; Safe Diet, U8959P Version 0156) or an isocaloric control diet (16.8% protein, 17.4% fat, 65.8% carbohydrate; Safe Diet, U8978P Version 0022) starting from day 2 of gestation and continuing through the lactation period. Food intake was monitored for 24 hours, and no difference was observed between the chow and low–protein diets. Female offspring were weaned and euthanized 22 days after birth.

### Rat pancreatic islet isolation

After sacrificing 22-day–old F1 progeny of control–chow diet (CD)-fed rats and 22-day–old F1 progeny of low–protein diet (LP)-fed–rats, pancreatic islets were isolated. Islets from the same dam were subdivided into two groups, one for extracting RNA from whole cell lysates (WCL) and the other for preparing mitochondrial fractions (MF). Rat pancreatic islets were isolated by collagenase digestion, purified on a Histopaque density gradient and handpicked as previously described.^20^

### Isolation of mitochondrial fractions

Mitochondrial fractions and whole–cell lysates were separated by working swiftly and maintaining all samples on ice throughout the procedure. Mitochondria were extracted from rat islets using a method previously established and thoroughly validated.^19^^,^^21^ Cells were harvested, rinsed three times with 1X PBS (137 mM NaCl, 8.1 mM Na_2_HPO_4_, 2.7 mM KCl, 1.47 mM KH_2_PO_4_, pH 7.4) and resuspended in 1 mL of 1X PBS. A 200 µL aliquot of the supernatant, containing whole islets, was then collected and mixed by vortexing for 30 seconds with 500 µL of Trizol, prior to RNA extraction as detailed in the “RNA extraction” section. The remaining 800 µL were centrifuged at 1000 × g for 5 minutes at 4 °C, after which the supernatant was carefully removed. The resulting pellet was resuspended in ice–cold mitochondrial isolation buffer (MIB), composed of 200 mM sucrose, 10 mM Tris/MOPS, and 1 mM EGTA/Tris. This buffer was filter–sterilized and stored at 4 °C (pH 7.4). The 1 M Tris/MOPS stock was prepared by dissolving 12.1 g of Tris base in 70 mL of water, adjusting the pH to 7.4 with MOPS, followed by filtration and storage at 4 °C. The 0.2 M EGTA/Tris buffer was made by dissolving 3.8 g of EGTA in 10 mL of water, then adding approximately 30-40 mL of 1 M Tris/MOPS until fully dissolved; it was filtered and stored at room temperature (target pH 6.7). The cell suspension was then sonicated in three 15-second bursts. To further disrupt cell membranes, the sample was drawn into a 1 mL syringe fitted with a 27-gauge needle and expelled forcefully against the wall of a 1.5 mL tube. This step was repeated three times. Samples were centrifuged at 600 × g for 5 minutes at 4 °C, and the resulting supernatant was transferred and centrifuged again at 10,000 × g for 5 minutes at 4 °C. The final pellet, representing the mitochondrial fraction (MF), was resuspended in 700 µL of Trizol and vortexed for 30 seconds prior to RNA extraction, as outlined in the “RNA extraction” section.

To validate the purity of the mitochondrial fraction, we used qRT–PCR to demonstrate enrichment of the mitochondrial tRNA fragment mt–tRF–Leu^TAA^, as described in the “tRF quantification by real–time PCR” section.

### RNA extraction

Isolated rat islets were washed twice with 1X PBS, resuspended in 700 µL QIAzol lysis reagent (Qiagen, #79306), and vortexed for 30 seconds and stored at −80 °C until RNA extraction. Total RNA was extracted using miRNeasy mini (Qiagen, #217004) or micro kit (Qiagen, #217084) and treated with DNAse (Promega, Cat#M6101).

### Small RNA–sequencing

Small RNA sequencing was conducted on pancreatic islets obtained from 22-day–old female rats that had been subjected to protein deficiency during both prenatal and postnatal development, compared to their corresponding control groups (GEO accession GSE303428). Total RNA was extracted using the miRNeasy micro Kit (Qiagen, #217084), and cDNA libraries were generated using the QIAseq miRNA NGS 48 Index IL Kit (Qiagen, #331502). Library preparation, sequencing, alignment, and read quantification were performed at the Genomic Technologies Facility (GTF) of the University of Lausanne. RNA libraries were size–selected for specific small RNA biotypes using an automated gel excision system. Library quality was assessed and quantified using the Agilent BioAnalyzer 2100, followed by sequencing on an Illumina HiSeq 2500 platform. Sequencing was carried out using a 50-base–pair single–read configuration, generating 145 million reads per sample. Data quality was assessed using FastQC software (version 0.10.1). Unique sequence counts were determined based on unique molecular identifiers (UMIs). microRNA annotation and differential expression analysis was performed using the RNA–seq analysis portal (GeneGlobe, Qiagen, https://geneglobe.qiagen.com/). We excluded all small non–coding RNAs with mean read counts < 13 total reads (lower limit of detection). Reads were mapped to the rat transcriptome, including mature tRNAs, microRNAs, piRNAs, rRNAs, snRNAs, and snoRNAs. tRFs were annotated by computational detection using the tRNA gene algorithm tRNAscan–SE used for the genomic tRF database (GtRNAdb). The mitotRNAdb database was used for annotation of mitochondrial tRFs. Normalization and differential expression analysis (False Discovery Rate ≤ 0.05) were performed using raw read counts as input using R package DESeq2.^22^ Estimation of dispersion performed using the negative binomial error model showed that total RNA samples behaved appropriately, with a good separation between groups. MicroRNA, piRNA and other small ncRNA mitochondrial enrichment analyzes were performed and normalized by dividing read counts for each microRNA, piRNA or small ncRNA in each sample by the total number of microRNA, piRNA or small ncRNA read counts in that sample.

### tRF quantification by real–time PCR

Quantification of the mitochondrially encoded tRNA–derived fragment mt–tRF–Leu^TAA^ by real–time PCR was carried out using the miRCURY LNA Universal RT microRNA PCR system, starting with 160 ng of total RNA (Qiagen, #339340 for RT kit and #339347 for Sybr Green). To calculate relative RNA levels between different samples, we employed the delta–delta Ct method, (2–∆∆Ct). The input sequence used for primer design of rno–mt–tRF–Leu^TAA^ was as follow: AAGACTTAAAACCTTGTTCCCAGAGGTTCAAATCCTCT.

### Mitochondria–enriched microRNA targetome analyzes

Predicted target genes of ten microRNAs that were enriched in the mitochondrial fraction and simultaneously reduced in the whole–cell lysate of LP–exposed rat islets were identified using TargetScan. These targetomes were then analyzed using Gene Ontology annotations and data from the Human Protein Atlas to identify biological pathways specifically associated with mitochondrial function. The analysis focused on pathways falling into eight major functional categories relevant to mitochondrial biology and indicated as color–coded columns.

### Quantification and statistical analysis

Data analysis as well as graphs and plots were performed in Excel, R package DESeq2 (version 4.2.0) and GraphPad Prism version 8.0.0 for Windows (GraphPad Software, San Diego, California USA, www.graphpad.com). To compare a data set to a control value set to 1, one–sample Student’s *t*-test was used. Statistical significance was considered whenever *p*-values were ≤ 0.05.

## Results

### Low–protein diet modulates small non–coding RNA expression in rat pancreatic islets

**Figure 1.**
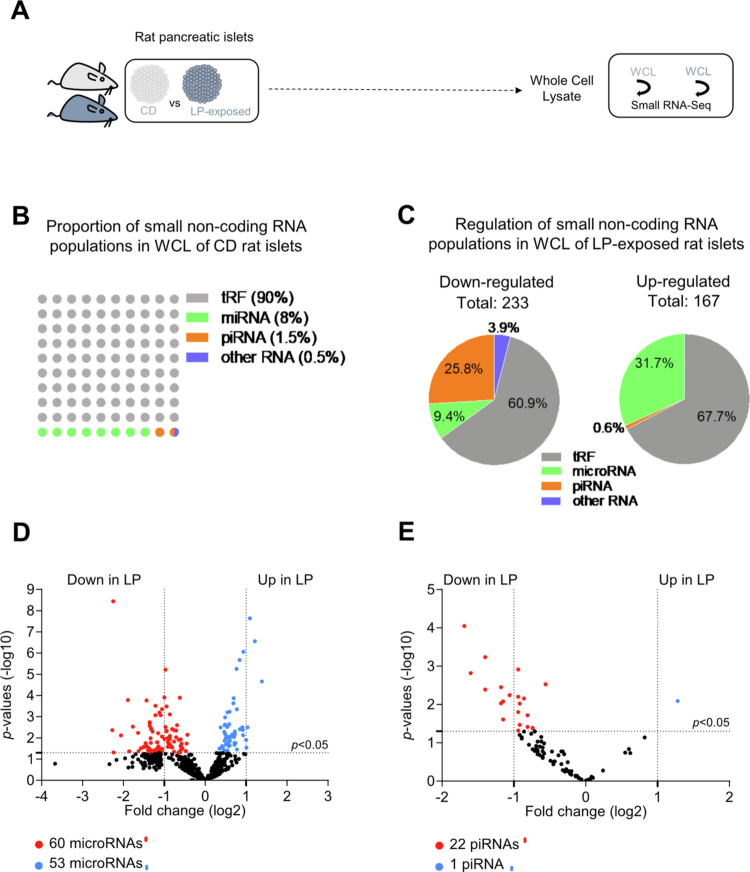
Changes in small ncRNA expression profile in rat pancreatic islets in response to LP diet exposure. (A) Schematic representation of the experiment. Pancreatic islets were isolated from 22-day–old F1 progeny of CD–fed rats and 22-day–old F1 progeny of LP–fed–rats. Small RNA sequencing was performed on WCL from four LP–exposed and four CD rats. (B) Dot plot showing the proportions of unique sequences corresponding to tRFs, microRNAs, piRNAs, and other small non–coding RNAs (including snRNAs and snoRNAs) in WCL of control rat islets. (C) Pi charts showing the classification of the 400 small ncRNAs significantly modulated (233 downregulated and 167 upregulated) amongst the 6857 small ncRNAs detected in pancreatic islets of LP–exposed rats. The figure shows the percentage of differentially expressed sequences in WCL of LP–exposed rat islets belonging to each of the four categories (tRFs, microRNAs, piRNAs, and other small ncRNAs (including snRNAs and snoRNAs)). Total read counts ≥ 13 cutoff, *p* ≤ 0.05. (D) Volcano plot showing the 113 microRNAs significantly changed amongst the 543 microRNAs detected in WCL of pancreatic islets of LP–exposed rats. Total read counts ≥ 13 cutoffs, *p* ≤ 0.05. (E) Volcano plot showing the 23 piRNAs significantly changed amongst the 118 piRNAs detected in WCL of pancreatic islets of LP–exposed rats. Total read counts ≥ 13 cutoffs, *p* ≤ 0.05.

To explore the impact of small non–coding RNAs in the development and progression of T2D, we analyzed how their expression patterns in pancreatic islets are altered under diabetes–prone conditions. To this end, we analyzed by small RNA–sequencing the levels of microRNAs, piRNAs, tRFs and other small ncRNAs in whole islet cells from 22-day–old F1 progeny of control–chow diet (CD)-fed rats and 22-day–old F1 progeny of low–protein diet (LP)-fed–rats during pregnancy and lactation (Figure 1A). The analysis of the RNA–sequencing data revealed that 45% of the reads were mapped to microRNAs, 24% to tRFs and less than 1% to piRNAs (GEO accession GSE303428). About 90% of the 6837 distinct small ncRNAs sequences identified were tRFs, 8% microRNAs, 1.5%, piRNAs and 0.5% other known small ncRNAs (Figure 1B). Among the detected small ncRNAs identified, 233 were decreased and 167 increased in response to protein deficiency (Figure 1C). Most of the downregulated small ncRNA sequences belonged to tRFs (60.9%), followed by piRNAs 25.8%, microRNAs 9.4%, and other small ncRNA species (3.9%) (Figure 1C, Supplementary Table 1). In comparison, the upregulated small ncRNAs sequences were primarily composed of tRFs (67.7%) and microRNAs (31.7%), with piRNAs representing only 0.6% (Figure 1C). The relative proportions of tRFs between control and LP–exposed rat islets remained largely consistent whereas microRNA, piRNA and other ncRNA populations exhibited greater divergence. Specifically, in response to protein deficiency during fetal and early postnatal life, 60 microRNAs were reduced, while 53 were upregulated (adjusted *p*-value ≤ 0.05) (Figure 1D). Concomitantly, 22 piRNAs were significantly downregulated, with only one showing an increase (adjusted *p*-value ≤ 0.05) (Figure 1E), highlighting a dynamic and selective reshaping of the islet small ncRNA landscape under nutrient stress.

### Fetal and postnatal protein deficiency reprograms mitochondrial small ncRNA landscape in rat pancreatic islets

**Figure 2.**
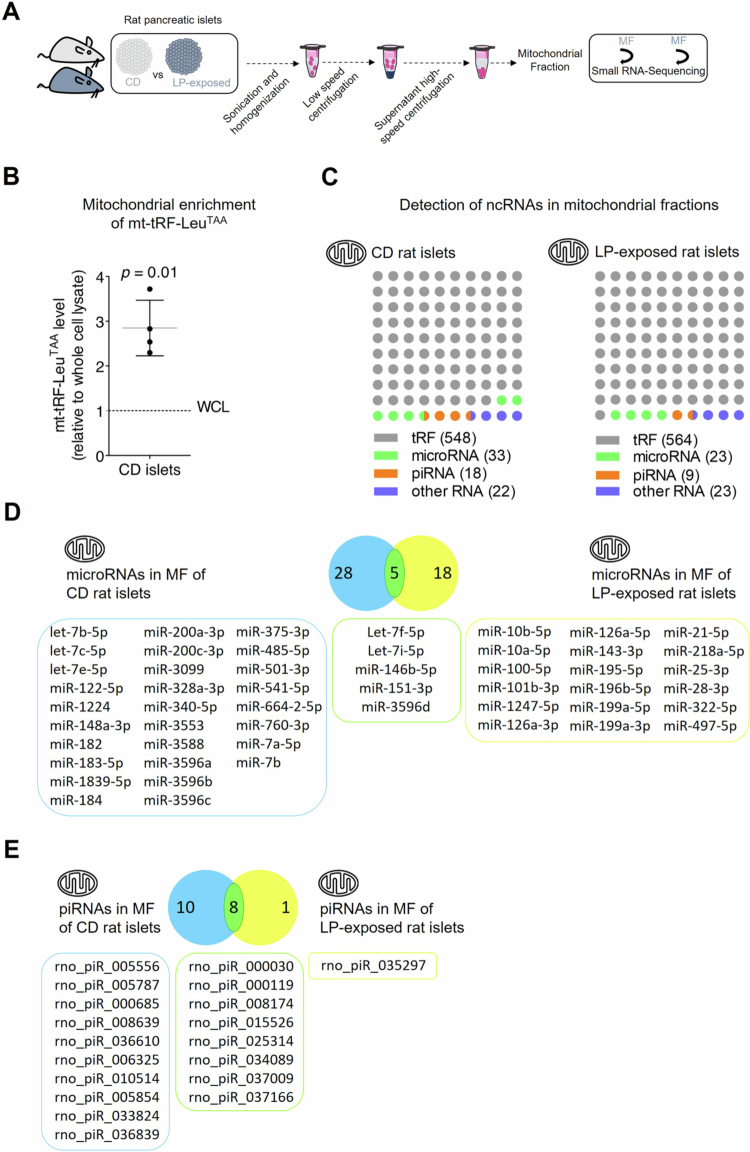
Differential mitochondrial RNA expression in rat pancreatic islets following exposure to a LP diet. (A) Schematic representation of the experiment. Pancreatic islets were isolated from 22-day–old F1 progeny of CD–fed rats and 22-day–old F1 progeny of LP–fed–rats. Small RNA sequencing was performed on MF from four LP–exposed and four CD rats. (B) Enrichment of the mitochondrial fragment mt–tRF–Leu^TAA^ in mitochondrial preparations of CD female rat islets. Data are expressed as fold change relative to whole cell lysate ± SD of 4 independent experiments, one–sample Student’s t–test. (C) Number of individual small ncRNA sequences significantly enriched in mitochondrial fractions of pancreatic islets subdivided by categories (tRFs, microRNAs, piRNAs and other ncRNA molecules (including snRNAs and snoRNAs)). *N* = 4 22-day–old F1 progeny of CD–fed dams vs *N* = 4 22-day–old F1 progeny of LP–fed dams. Total read counts ≥ 13 cutoff. **(D-**E) Venn diagram showing 51 microRNAs (E) and 19 piRNAs (F) enriched in MF of pancreatic islets of CD and LP–exposed rats. Common hits that overlap between the two groups (5/51 microRNAs and 8/19 piRNAs) are highlighted in green. *N* = 4 22-day–old F1 progeny of CD–fed rats and *N* = 4 22-day–old F1 progeny of LP–fed rats. ≥ 2-fold enrichment and total read counts ≥ 13 cutoffs, *p* ≤ 0.05.

Maternal protein restriction during pregnancy drives early mitochondrial dysfunction in pancreatic islets, which underlies the long–term metabolic disturbances observed in offspring. Despite growing evidence that ncRNAs are key players in mitochondrial dysfunction in these animals, the global profile and role of mitochondrial ncRNAs in protein–restricted offspring remains undetermined. To this end, we analyzed by small–RNA sequencing the mitochondrial small ncRNA transcriptome. We compared levels of tRFs, microRNAs, piRNAs, and other small ncRNAs in isolated mitochondria from 22-day–old offspring of rats fed either a CD or a LP diet during pregnancy and lactation (Figure 2A). In our prior work, we demonstrated that a mitochondrially–encoded tRF, mt–tRF–Leu^TAA^, is roughly ten times enriched in mitochondrial fractions compared to whole–cell lysates in both human and rodent islet cells.^19^ Consistent with these findings, we confirmed enriched mt–tRF–Leu^TAA^ levels in the mitochondrial fractions subjected to RNA sequencing in the current study (Figure 2B).

Systematic analysis of the RNA–sequencing data revealed that most of the individual sequences identified in mitochondria are tRFs, followed by microRNAs, piRNAs, and other small ncRNAs. Indeed, we detected 548 tRFs, 33 microRNAs, 18 piRNAs, and 22 other small ncRNAs enriched in mitochondrial extracts from control rat pancreatic islets (GEO accession GSE303428) (Figure 2C, Supplementary Table 2). The relative proportions of tRFs, microRNAs, piRNAs and other ncRNAs between mitochondria of control and LP–exposed rats remained largely stable (Figure 2C). In fact, we observed a similar number of tRFs, microRNAs, piRNAs, and other small ncRNAs enriched in mitochondrial fractions of rats exposed to a LP diet. However, maternal protein deficiency during pregnancy and lactation induced a marked alteration in the mitochondrial small ncRNA profile of 22-day–old offspring. Notably, of the 33 microRNAs enriched in the mitochondrial fractions of pancreatic islets from control rats and the 23 detected in those from LP–exposed rats, only 5 were shared between the two groups (Figure 2D). These results indicate a substantial reprogramming in mitochondrial microRNA composition in response to early–life nutritional stress. Protein restriction during fetal and postnatal development altered the mitochondrial piRNA expression profile to a much lesser extent than that of microRNAs. In the mitochondrial fractions of pancreatic islets, 18 piRNAs were enriched in control rats and 9 in LP–exposed rats, with 8 piRNAs shared between the two groups, indicating a moderate shift in mitochondrial piRNA composition following protein restriction (Figure 2E). In light of these findings, we focused our investigation on LP–induced shifts in mitochondrial microRNA composition.

### Nutritional stress in LP–exposed islet cells caused microRNA redistribution with concomitant cytosolic reduction and mitochondrial enrichment of microRNAs

**Figure 3.**
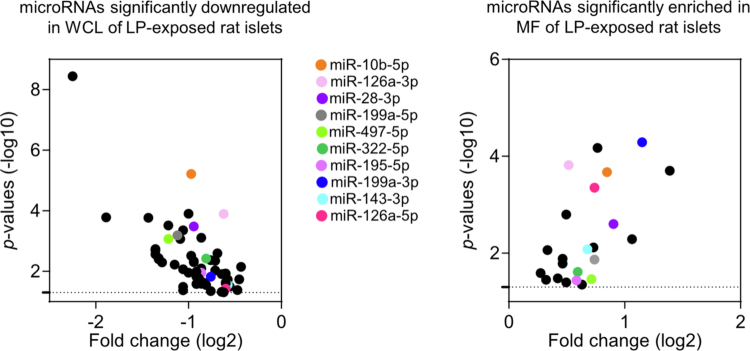
MicroRNAs downregulated in whole cell lysates and enriched in mitochondrial fractions of pancreatic islets following LP exposure. Left panel, Zoom in of the volcano plot Figure 1D for the 60 microRNAs that display a significant downregulation in WCL of pancreatic islets of LP–exposed rats. Right panel, Volcano plot showing of the 18 microRNAs that display a significant enrichment in MF of pancreatic islets of LP–exposed rats presented in Figure 2D. Middle panel, List of the ten microRNAs that display a reduction in whole islet cells from LP–exposed rats and a concomitant accumulation in mitochondria of islet cells from these animals are color–coded. Total read counts ≥ 13 cutoff. The dotted lines indicate the significant changes with *p* ≤ 0.05.

We observed that among the 60 microRNAs significantly downregulated in islet cell lysates of LP–exposed rats (Figure 3 left panel), ten were simultaneously enriched in the purified mitochondrial fractions (Figure 3 right panel), suggesting a targeted redistribution toward the mitochondrial compartment under nutritional stress.

### LP–induced mitochondrial localization of specific microRNAs potentially regulates core mitochondrial genes

**Figure 4.**
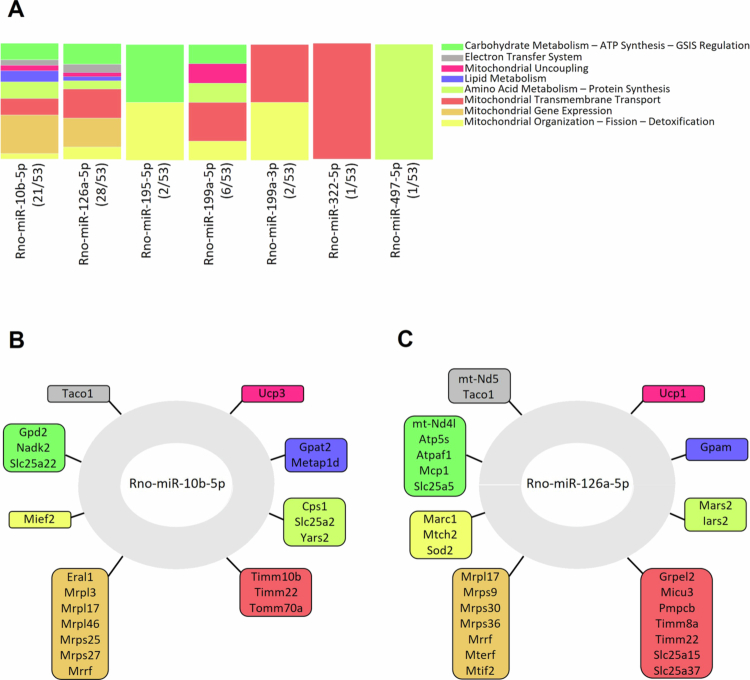
Predicted networks regulated by mitomiRs involving mitochondrial–and nuclear–encoded genes essential for mitochondrial function. (A) The target genes of identified microRNAs enriched in MF of LP–exposed islet cells. Genes are color–coded based on the Gene Ontology Pathways they are associated with. Of the ten mitomiRs simultaneously reduced in WCL and enriched in MF of LP–exposed islets, seven are predicted to target 53 genes linked to mitochondrial function and localized to mitochondria. The number of genes targeted by each of the seven microRNAs is indicated in brackets. (B) Target genes of rno–miR-10b-5p linked to mitochondria, categorized by Gene Ontology pathways. (C) Mitochondria–associated targets of rno–miR-126a-5p, organized according to Gene Ontology classifications.

Based on miRNA–target gene interaction predictions (TargetScan), we conducted a Gene Ontology pathway analysis and examined which biological pathways were affected with the ten identified mitomiRs that were decreased in total cell content and increased in mitochondria of LP–exposed rat islets. This analysis highlighted enrichment of biological pathways crucial for mitochondrial metabolism–secretion coupling as well as mitochondrial function, such as nutrient metabolism, electron transfer system, ATP synthesis, GSIS regulation and mitochondrial organization. Notably, rno–miR-10b-5p and rno–miR-126a-5p are both predicted to target roughly half of the genes that contributed to the identification of the enriched pathways, suggesting their potential central role in regulating these biological processes (Figure 4A).

Using miRNA–target prediction tools, we constructed mRNA–miRNA interaction networks for rno–miR-10b-5p and rno–miR-126a-5p. The former is predicted to exclusively target nuclear–encoded genes critical for mitochondrial function and metabolism, including Gpd2, Nadk2, and several mitochondrial transmembrane transporters involved in anterograde and retrograde signaling (Figure 4B). In contrast, rno–miR-126a-5p is predicted to target both mitochondrially–encoded genes, such as mt–Nd5 and mt–Nd4l, and nuclear–encoded genes, including Sod2, Atp5s, Mcp1, and Slc25a5, all of which play essential roles in mitochondrial homeostasis (Figure 4C).

## Discussion

Maternal protein deficiency induced a marked remodeling of the small ncRNA landscape in pancreatic islets of neonatal rats, with coordinated changes evident at both whole–cell and mitochondrial levels. This reconfiguration points to ncRNAs, particularly tRFs and microRNAs, as central mediators of the islet’s adaptive response to early–life nutrient stress.

Under normal conditions, tRF sequences are abundant in the islet ncRNA population, consistent with their emerging role as regulators in metabolically active tissues. While tRFs were also the most affected class under low–protein (LP) exposure, their detailed mitochondrial profiling was not pursued here as we have previously characterized the mitochondrial tRF signature, including mtDNA–encoded tRFs.^19^ The LP diet altered the expression of nearly 400 small ncRNAs, with microRNAs and tRFs most dynamically regulated. These changes were not random but appeared selective, as the relative abundance of tRFs remained stable whereas microRNA and piRNA populations shifted more substantially. The upregulation of miR-375, also reported in similar nutritional models,^15^ is particularly noteworthy. Like rno–miR-126a-5p identified here, miR-375 is predicted to target ATP synthase, and its established role in regulating *β*-cell proliferation and insulin secretion suggests it may act in concert with specific mitomiRs to impair *β*-cell function in LP offspring.

Mitochondrial profiling revealed that tRF sequences were again the most numerous, followed by microRNAs, piRNAs, and other ncRNAs. Enrichment of a mtDNA–encoded tRNA in mitochondrial fractions relative to whole–cell lysates confirmed sample purity. Despite similar proportional representation, the mitochondrial composition of the ncRNAs was markedly altered in LP offspring, with only a small fraction of mitochondrial microRNAs shared between groups. This selective reorganization aligns with evidence that mitochondrial small RNA profiles are highly dynamic and sensitive to cellular and environmental context.^23^ Importantly, several microRNAs downregulated in whole cells were concomitantly enriched in mitochondria, suggesting active redistribution toward this organelle under nutrient stress.

Functional analysis indicated that these redistributed microRNAs regulate genes essential for mitochondrial metabolism–secretion coupling, ATP synthesis (Gpd2, Nadk2, Slc25a22, mt–Nd4l, Atp5s, Atpaf1, Mcp1, Slc25a5), electron transport (mt–Nd5, Taco1), and structural organization (Mief2, Marc1, Mtch2, Sod2). Many target nuclear–encoded transcripts known to be imported into mitochondria, where they can be locally regulated, and several encode mitochondrial transmembrane proteins–consistent with reports that imported microRNAs localize to the mitochondrial surface or intermembrane space.^24^ Among the most relevant candidates, miR-10b-5p and miR-126a-5p emerged as central regulators. MiR-10b-5p depletion leads to *β*-scell degeneration and has been implicated in diabetes–associated tyrosine kinase and estrogen signaling pathways.^25^^,^^26^ MiR-126a-5p shows context–dependent changes in metabolic disease, being reduced in diabetic rat hearts but elevated in vascular smooth muscle cell dysfunction in type 2 diabetes.^27^^,^^28^ Other identified mitomiRs also carry strong functional associations: miR-199a-3p promotes *β*-cell apoptosis,^29^ whereas miR-322 confers cardioprotection in high–fat diet–fed mice via mitochondrial pathways^30^ yet suppresses insulin secretion in *β*-cells through repression of *Stxbp1.*^31^ Collectively, these profiles suggest that mitomiRs in LP–exposed islets may coordinate adjustments in mitochondrial bioenergetics, redox handling, *β*-cell survival, and insulin secretory capacity.

The mitochondrial alterations observed here are unlikely to be restricted to islets. Comparable structural and functional defects have been reported in skeletal muscle^32^ and kidney following prenatal protein restriction,^33^ supporting the concept of a systemic developmental reprogramming of mitochondrial biology that increases metabolic vulnerability later in life.

In conclusion, maternal protein restriction reconfigures the small ncRNA repertoire of pancreatic islets and promotes the mitochondrial redistribution of a distinct set of miRNAs with predicted roles in energy metabolism, mitochondrial organization, and *β*-cell function. These findings provide a mechanistic link between early–life nutrient status, mitochondrial regulation, and long–term susceptibility to type 2 diabetes. Future studies should determine whether these ncRNA shifts are adaptive or maladaptive, define their persistence into adulthood, and assess their potential as biomarkers or therapeutic targets in the developmental origins of metabolic disease.

## Supplementary Material

Revised_Supplementary Table 1.xlsx Revised_Supplementary Table 1.xlsx

Revised_Supplementary Table 2.xlsx Revised_Supplementary Table 2.xlsx

## Data Availability

Sequencing data are available on Gene Expression Omnibus under the accession number GSE303428.
